# Towards a Non-Biased Formaldehyde Quantification in Leather: New Derivatization Conditions before HPLC Analysis of 2,4-Dinitrophenylhydrazine Derivatives

**DOI:** 10.3390/molecules25235765

**Published:** 2020-12-07

**Authors:** Caroline Bourgeois, Nicolas Blanc, Jean-Claude Cannot, Claire Demesmay

**Affiliations:** 1 CTC, Comité Professionnel de Développement Economique, Cuir, Chaussure, Maroquinerie, Ganterie, 4 Rue Hermann Frenkel, 69007 Lyon, France; nicolas.blanc@ctcgroupe.com (N.B.); jccannot@ctcgroupe.com (J.-C.C.); 2 CNRS, Institut des Sciences Analytiques, Université Claude Bernard Lyon 1, UMR 5280, 5 Rue de la Doua, 69100 Villeurbanne, France; claire.demesmay-guilhin@univ-lyon1.fr

**Keywords:** formaldehyde, leather, derivatization, DNPH derivatives, liquid chromatography

## Abstract

In leathers, formaldehyde is currently analyzed according to EN ISO 17226-1 standard, by reversed phase liquid chromatography after off-line precolumn derivatization with 2,4 dinitrophenylhydrazine (DNPH) in strong acidic conditions. We first demonstrate that this standard is not adapted to leather retanned with resins likely to release formaldehyde by hydrolysis. Indeed, formaldehyde content may be largely overestimated due to concomitant resin hydrolysis (in harsh acidic conditions) that releases formaldehyde during the derivatization step and during the waiting time on autosampler before analysis. Therefore, we thoroughly studied the derivatization step in order to propose new derivatization conditions. Replacing orthophosphoric acid by less acidic buffer solutions is not enough to avoid hydrolysis. A derivatization without adding acid is realized by solubilizing DNPH in acetonitrile instead of orthophosphoric acid. These conditions lead to a complete derivatization of formaldehyde in 3 h at 50 °C (in a water bath) while avoiding the hydrolysis of co-extracted dicyandiamide and melamine resins. The as-obtained leather extracts are stable over time. Formaldehyde contents found with this method agree with the formaldehyde content measured immediately at the end of derivatization reaction in standard conditions or with formaldehyde content measured by a home-designed flow injection analysis with acetylacetone online derivatization and UV detection.

## 1. Introduction

The European commission regulation No 605/2014 classifies formaldehyde as a class 1B carcinogen. Indeed, formaldehyde is dangerous: it is flammable, irritating for the respiratory tract, skin, eyes [1], and may cause an allergic skin reaction [2]. Despite its dangerousness, formaldehyde is used in various industrial fields such as the wood [3], cosmetic [2], textile [4], and leather industries [5,6,7]. In leather, formaldehyde was previously used for tanning or finishing because of its crosslinking properties [8]. Nowadays, it is mainly used to synthetize quick and easy to use tanning agents (such as dicyandiamide and melamine resins) that give suitable properties to leather [9]. Such dicyandiamide and melamine resins are synthetized by C-N bond formation during polycondensation of dicyandiamide and melamine with formaldehyde. This reaction is known to be reversible: hydrolysis (for example consecutive to rain or perspiration [5] can cleave these bonds and releases formaldehyde by nucleophilic substitution reaction [5,8]. This releasable formaldehyde is considered as free formaldehyde, which means it is not leather bound to any degree [10]. Therefore, formaldehyde can be found in the leather final product (resins used as tanning agents may contain residual free formaldehyde) but it can also be released during the use of the final leather product.

In leather, formaldehyde could generate allergic skin reaction by skin contact [2]. That is why there are various regulations concerning formaldehyde in leather depending on country and final use. For example, in China (GB 20400), the formaldehyde concentration limit is 20 ppm in leather for children products, 75 ppm for products in direct contact with skin and 300 ppm for products without skin contact. In Europe, there is currently no concentration limit for formaldehyde in leather except for toys, starting from May 2021. For leather or textile toy materials, the European Commission has set the formaldehyde content limit at 30 ppm (COMMISSION DIRECTIVE (EU) 2019/1929). 

Therefore, it is of paramount importance to have an analytical method to reliably determine the formaldehyde content in leather. To check regulatory requirements, the EN ISO 17226-1 Standard is the Official Method in the case of leather. It is an analytical method for measuring free formaldehyde and released formaldehyde in leather. The formaldehyde content measured with this standard is defined as “the quantity of free-formaldehyde and formaldehyde extracted through hydrolysis contained in a water extract under standard conditions”. This standard is divided into two parts that aim at measuring formaldehyde in leather samples and should give the same “trend”. In both parts, the initial phase of extraction of formaldehyde present in leather is the same and it is carried out with a suitable detergent solution at 40 °C (1 h stirring in a water bath). The temperature and duration of this extraction step have to be respected because an increase in temperature and/or in time triggers an increase in formaldehyde content [8,11]. The physical state of the sample has also to be strictly controlled as it may have an influence on the formaldehyde content measured [11]. The two parts of the standard implement different quantification procedures to quantify the released formaldehyde. The part 2 is the oldest one whereas the part 1, the latest one, should be used in the case of dispute. In part 2, formaldehyde is off-line derivatized with acetylacetone in mild acidic conditions and the obtained derivative is directly analyzed by colorimetry at 412 nm without any separation step. Some dyes may trigger interferences [12]. Moreover, free formaldehyde can be generated during the heating step required for the derivatization procedure and derivatives are not stable (photodegradable) and should be analyzed immediately.

In part 1, the quantification of the formaldehyde extracted from leather is carried out by reversed phase HPLC-UV after an off-line pre-column derivatization with 2,4-dinitrophenylhydrazine (DNPH). In this part, spectrophotometric interferences can be avoided. Indeed, the off-line pre-column derivatization with DNPH is followed by a liquid chromatographic separation. The introduction of a separation step alleviates spectrophotometric interferences and allows decreasing the limit of quantification [13,14,15] when compared with part 2. Regarding the chromatographic step of part 1, the standard specifies that samples must be analyzed by chromatography one hour after the beginning of the derivatization (probably for derivatization kinetic purposes) but not more than three hours after the beginning of the derivatization. According to Wu et al. [16] derivatives need to be analyzed soon after reaction due to instability of derivatives. According to Cuadros et al., the hydrolysis of coextracted resins could occur, which could trigger an increase of formaldehyde concentration [17]. Indeed, samples analyzed at the end of the sequence usually present a higher formaldehyde content [17]. According to these authors, some resins (like melamine or dicyandiamide) can be co-extracted during the extraction step and then they can release formaldehyde by hydrolysis over time [17]. For example, for a leather tanned with chromium and retanned with various products including melamine resin, these authors reported a formaldehyde concentration increase of about 25% between extreme recommended analysis time-frame (60 and 180 min) [17]. 

Considering the chromatographic analysis duration is about 10 min, no more than 15 samples can be analyzed during these 2-h periods of the standard, thus limiting the number of samples to be analysed in one routine sequence. Moreover, one might legitimately wonder about the selection of this specific time frame and about the accuracy of the obtained results. Being stricter on setting the reaction time between dinitrophenylhydrazine and formaldehyde extracted from leather, as recommended by Cuadros [17], does not appear sufficient to ensure the accuracy of the results.

In order to measure non-biased formaldehyde concentrations in leathers (including leathers retanned with formaldehyde-based resins), the standard 17226-1 should be modified with an analytical method more reliable whatever leather preparation. Considering that resins are probably hydrolysed in the acidic media used during the derivatization step, the derivatization conditions have to be modified to avoid such hydrolysis.

The first purpose of this study is to demonstrate that an accurate and non-biased determination of formaldehyde in leathers is not possible with the actual standard EN ISO 17226-1 even in the recommended time-frame and that the hydrolysis of co-extracted resins is responsible for the overestimation of formaldehyde content. New derivatization conditions are then explored to ensure a quantitative and reproducible derivatization of formaldehyde with DNPH while suppressing the hydrolysis of co-extracted resins. The accuracy of the obtained results is then demonstrated by using an original home-designed FIA-UV method with an on-line derivatization with acetylacetone. 

## 2. Results and Discussion

### 2.1. Analysis of Samples Based on EN ISO 17226-1 Standard Conditions: Influence of the Time Frame Between Derivatization and Analysis on the Formaldehyde Content

In the standard EN ISO 17226-1, samples are derivatized in a strong acidic media (with DNPH solubilized in orthophosphoric acid) immediately after the extraction step. According to this standard, the recommended time frame within they have to be analysed by reversed phase liquid chromatography is set between 1 h and 3 h after the beginning of the derivatization. 

In order to study the influence of this time frame (sample waiting time on the autosampler tray), formaldehyde was analyzed in leather samples immediately after preparation of the derivatization flask, and then repeatedly during the time-frame allowed by the standard (1 to 3 h) and also after a longer period of time. Three different leather samples were selected for this study: all of them were tanned with Cr III but they were subjected to different retanning protocols. Two of them (B and C) were retanned with formaldehyde-based resins, whereas the third one (A) was retanned without any resin. These two resins were selected due to their ability to release formaldehyde by hydrolysis. 

**Figure 1:**
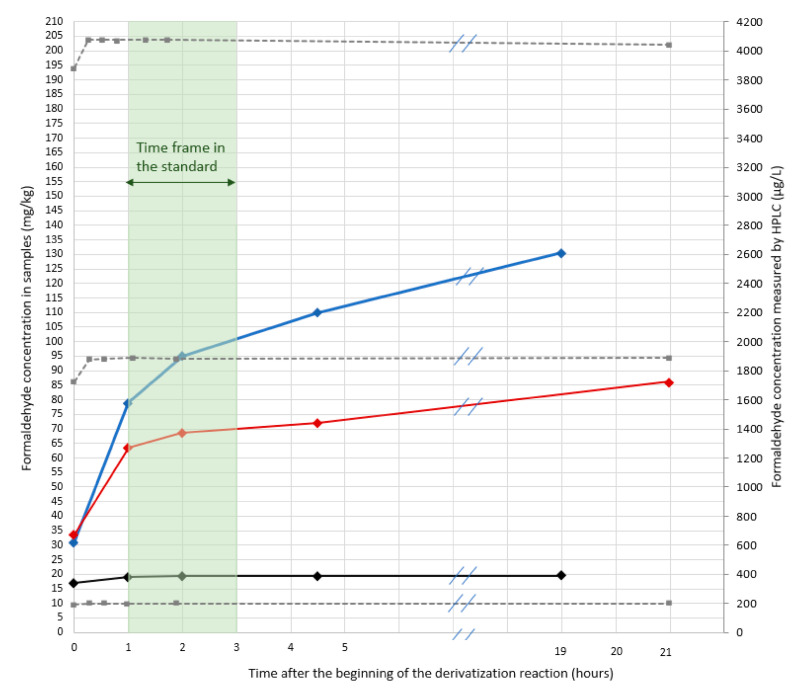
Formaldehyde content over the DNPH derivatization reaction time for lamb leathers tanned with chrome and retanned without resin (in black), with dicyandiamide resin (in blue) or with melamine resin (in red). Samples: prepared, extracted and derivatized following EN ISO 17226-1 standard conditions and analyzed at various time. The green area: the time frame allowed by the standard to analyze samples after their derivatization. Kinetic study of derivatization reaction with quality controls (QC) 200 µg L^−1^, 2000 µg L^−1^ and 4000 µg L^−1^ (in grey dotted lines).

As can be observed in Figure 1, and as expected, the measured formaldehyde content is higher for the two leathers retanned with resins than for the one retanned without resin (resins contain residual free formaldehyde). One may also observe that for the two leathers retanned with resins, the formaldehyde content continuously increases over time (even during the period allowed by the standard i.e., between 1 h and 3 h after the beginning of the derivatization reaction), whereas it is stable for the leather retanned without any resin. This increase (during the time frame recommended by the standard) is limited to about 10% for the leather retanned with melamine resin but is more pronounced and reaches 25% for the leather retanned with dicyandiamide resin. If the time spent on the autosampler tray further increases, the amount of formaldehyde increases too. 4.5 h after the beginning of the derivatization reaction (it is like the sample is at the end of a 4.5 h sequence of about 30 samples), formaldehyde content measured for the leather retanned with dicyandiamide resin is 30% higher than the one measured three hours earlier. Conversely, the formaldehyde content measured for the leather retanned without any resin is stable over time. If no issues are observed for the extracts from leathers retanned without resins, results obtained for leathers retanned with resins are open to dispute: the formaldehyde content measured according to the standard greatly depends on the time at which measurements are realized. And even more worrying, is the great increase between 0 and 1 h of derivatization for the two leathers retanned with resins: about +100% for the melamine-retanned leather and up to +160% for the dicyandiamide-retanned leather. Such an increase may be related to the progressive hydrolysis of co-extracted resins since it only occurs for leathers retanned with resins. These results suggest that the standard is not adapted for the determination of formaldehyde in leathers retanned with resin and that a strict control of the time at which measurements are realized is not enough to guarantee an accurate determination of formaldehyde.

It was first verified that the variation of the formaldehyde content during the first hour was not related to kinetic issues (incomplete reaction) for the leathers containing higher amounts of formaldehyde (those for which a great increase is observed over time). Standard solutions at 200 µg L^−1^, 2000 µg L^−1^ and 4000 µg L^−1^ of formaldehyde were prepared in the extraction media and then subjected to derivatization and chromatographic analysis. Standard solutions were analyzed immediately after preparing the derivatization flask (sample injected immediately after preparing the derivatization flask) and over time (until 19 h after the beginning of the reaction time).

The results (Figure 1) clearly show that whatever the concentration of the quality controls ranging between 200 to 4000 µg L^−1^ the derivatization is complete in less than 15 min and the amount of formaldehyde quantified is independent of the time laps between derivatization and quantification. Results also demonstrate that no degradation of the derivatives occurs as mentioned earlier in the literature [13]. So, the increase of formaldehyde content previously observed in some resin-retanned leathers during the waiting time on the autosampler tray is related to the presence of resins. These results agree with the trends described by Cuadros et al. [17]. In order to further this assumption, other experiments were carried out by simulating extraction and derivatization with resins.

### 2.2. Quantification of Formaldehyde in Dicyandiamide and Melamine Resin Pseudo-Extracts

The formaldehyde content in the formaldehyde-based resins used was determined by an adaptation of the EN ISO 17226-1. A given amount of formaldehyde-based resin (0.04 g) was pseudo-extracted with sodium dodecyl sulphate solution. Once extracted and after filtration, an aliquot of the filtrate (pseudo-extract) was reacted with dinitrophenylhydrazine and, afterwards, the derivative was quantified by HPLC. Chromatographic analyses were realized immediately after preparing the derivatization flask and over the time.

**Figure 2:**
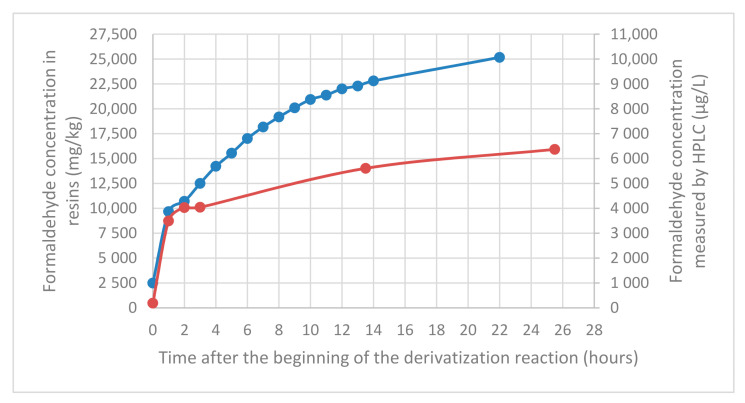
Formaldehyde content in dicyandiamide resin (in blue) and in melamine resin (in red) over the time. Resins (0.04 g) prepared, extracted (50 mL of 0.1% dodecane-1-sulfonic-acid aqueous solution) and derivatized following standard conditions used for leather (EN ISO 17226-1) and analyzed at various time.

For both resins, formaldehyde concentration in pseudo-extracts increases over time especially for dicyandiamide resin (Figure 2). However, it is not a kinetic problem, as seen previously, the reaction is almost immediate. Consequently, the increase is only due to the resin hydrolysis in pseudo-extracts over time and the subsequent derivatization of the formaldehyde released. The hydrolysis is very rapid during the first hour and continues for 24 h. 

To guarantee a reliable determination of the formaldehyde content in leathers, it is necessary to find derivatization conditions limiting the hydrolysis of the resins. As trends were similar for melamine resins and dicyandiamide ones, only dicyandiamide resins were studied thereafter.

### 2.3. New Derivatization Conditions for the Analysis of Formaldehyde in Dicyandiamide Resin

New conditions for DNPH derivatization reactions are necessary as being stricter on setting the reaction time is not enough to guarantee an accurate value of the formaldehyde content. The goal is to avoid resin hydrolysis in order to have a formaldehyde content as defined in EN ISO 17226-1 standard (formaldehyde content is the addition of “free formaldehyde” and “formaldehyde hydrolysed during extraction to produce free formaldehyde”) and this content has to be stable over time. In the EN ISO 17226-1 standard conditions, the DNPH is in an orthophosphoric acid solution. Harsh acidic conditions (the pH in the derivatization flask is about 1.3) are known to catalyse the derivatization reaction with DNPH but also to favour resin hydrolysis [18]. The first alternative consists in using less acidic and/or buffered media. Buffered conditions could be valuable to avoid pH changes that could take place with some leather extracts differing in pH. Therefore, buffered solutions were first evaluated as derivatization media. Since DNPH is not soluble in water, it was first solubilized at high concentration in acetonitrile (stock solution) and then a small aliquot of this stock solution was mixed with buffered solutions. Another alternative evaluated was to work in non-buffered or acidified solutions (buffer replaced by water). 

**Figure 3:**
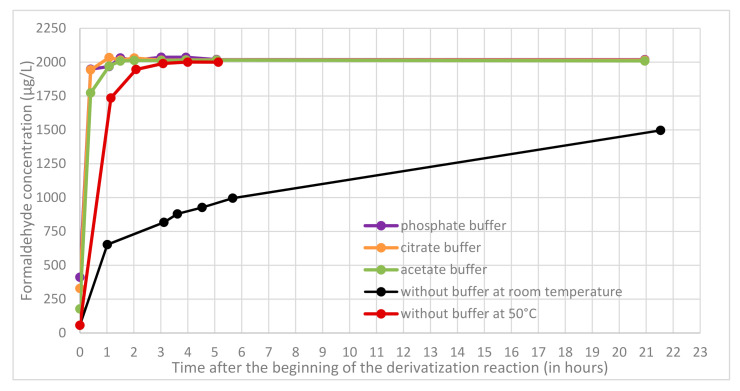
Reaction kinetic on 2000 µg L^−1^ quality control for different derivatization conditions: in purple with phosphate buffer, in orange with citrate buffer, in green with acetate buffer and without adding acid in black (at room temperature) and in red (at 50 °C in a water bath).

First, the kinetics of derivatization step was studied at high concentration level (2000 µg L^−1^) with quality control prepared in the extraction medium. In buffered acidic conditions (phosphate, citrate, and acetate buffer) respectively at pH 2.12, 3.13, and 4.75 the reaction required about 1 to 1.5 h to be complete and the derivative was stable over a long period (at least 21 h). In Figure 3, no significant difference in derivatization kinetics was observed for pH ranging from 2.12 to 4.75. Without any acidification, the reaction required about 2–3 days for completion at room temperature but could be catalysed by heating at 50 °C (with a water bath) to be complete in only 3 h. Following table sums up the different reaction times required to complete the derivatization reactions in all conditions tested (Table 1). 

These optimized derivatization conditions were then tested for the dicyandiamide resin and the results compared with those obtained in harsh acidic conditions. Indeed, our objective was to find conditions for which the derivatization is rapid and for which the waiting time before the chromatographic analysis has no influence on the final formaldehyde content measured. For each condition tested, formaldehyde was analyzed in pseudo-extracts of dicyandiamide resins immediately at the end of the derivatization reaction (just after the time required to complete reaction) and after various waiting times on the tray in order to see the influence on resin hydrolysis. All conditions were assayed the same day. It means derivatizations were realised on different aliquots of the same resin pseudo-extract. 

**Figure 4:**
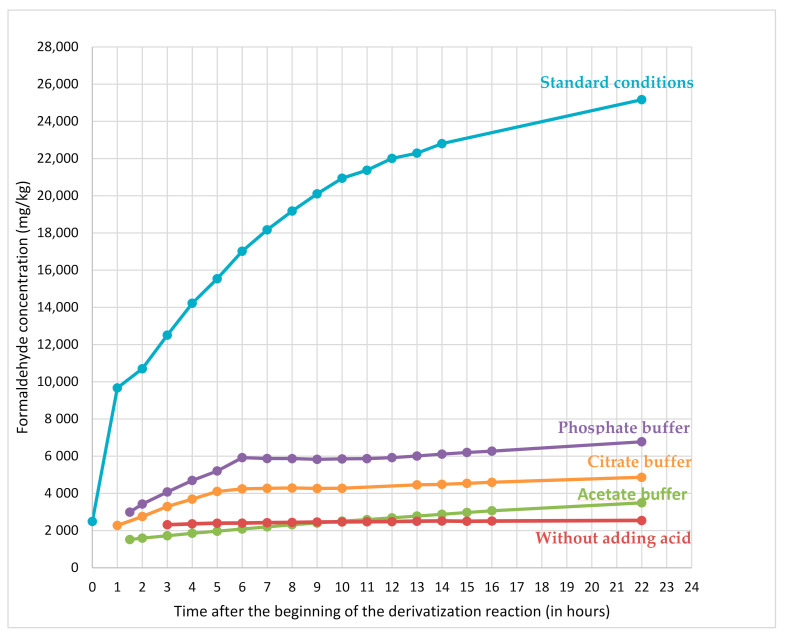
Formaldehyde content measured in dicyandiamide resin pseudo-extracts over derivatization reaction with DNPH in different derivatization conditions: in blue in standard conditions (EN ISO 17226-1), in purple with phosphate buffer, in orange with citrate buffer, in green with acetate buffer and in red without adding acid.

Decreasing the pH of the derivatization medium allows reducing hydrolysis whatever the conditions tested (Figure 4). The increase of buffered solution pH triggers a decrease of resin hydrolysis during derivatization and a decrease of formaldehyde content at the end of derivatization reaction. Regarding the evolution over time of the formaldehyde content determined in dicyandiamide resin pseudo-extracts (Figure 4), it increases for all conditions with acidic buffers (thus indicating that hydrolysis of the resin still occurs during the waiting time). Conversely, derivatization conditions without adding any acid gives a stable formaldehyde concentration whatever the waiting time.

**Figure 5:**
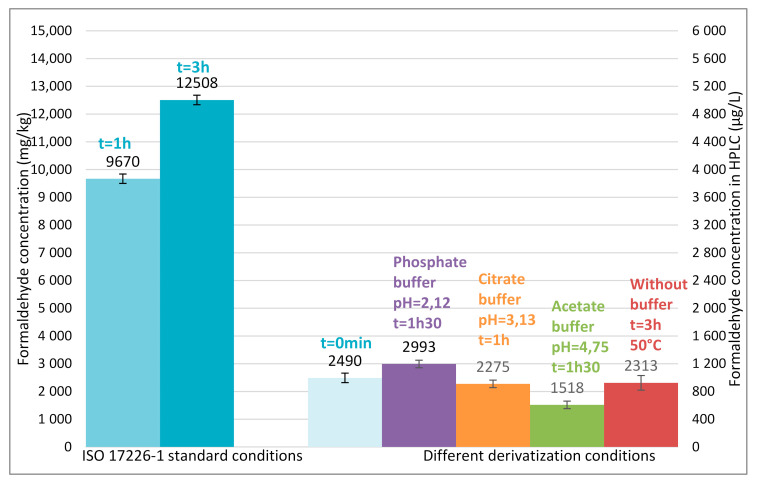
Formaldehyde content (residual free formaldehyde contained in the resin and formaldehyde resulting from resin hydrolysis) for dicyandiamide resin (0.04 g in 50 mL of 0.1% dodecane-1-sulfonic-acid aqueous solution) obtained at the end of reaction in different DNPH derivatization conditions. Uncertainties given as two standard deviations (calculated with three measurements: three independent derivatizations).

Figure 5 shows that the formaldehyde content (9670 mg/kg) measured in standard conditions (harsh acidic conditions) after 1 h of derivatization (beginning of the recommended time frame) is 4 times as much as the formaldehyde content (2490 mg/kg) determined immediately at the end of the derivatization reaction (the reaction is complete in few minutes). For non-buffered conditions at 50 °C, the amount of formaldehyde determined in resins is of the same order of magnitude than the one determined in buffered conditions or in harsh acidic conditions immediately after preparing the derivatization flask.

As a conclusion, buffered and less harsh acidic conditions did not succeed in avoiding resin hydrolysis even if it was less pronounced. Conversely, conditions with DNPH solubilized in acetonitrile (and without adding any acid) succeeded in avoiding resin hydrolysis: the formaldehyde content measured over time did not vary. Consequently, in these conditions, samples could be analysed few hours after their preparation without any bias related to the waiting time. 

### 2.4. Determination of Formaldehyde in Leathers by New Derivatization Conditions 

**Figure 6:**
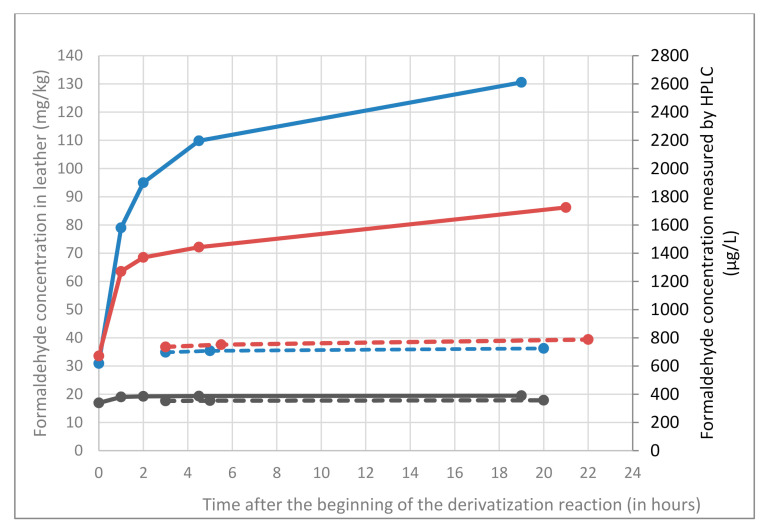
Formaldehyde content over DNPH derivatization reaction time; two derivatization conditions used: EN ISO 17226-1 standard conditions (solid lines), DNPH in acetonitrile at 50 °C during 3 h (dashed lines); lambs tanned with chrome and retanned without resin (in black) or with dicyandiamide resin (in blue) or melamine resin (in red).

The three leathers (containing or not resins) were analyzed with this new derivatization method (3 h in a water bath at 50 °C with DNPH in acetonitrile). Results measured over time (dashed lines), are compared with results initially obtained with harsh acidic conditions (solid lines) of EN ISO 17226-1 standard (Figure 6).

Comparable results are found for the three leathers with the new method and with EN ISO 17226-1 standard conditions directly after the beginning of the derivatization reaction. Contrary to standard conditions, for leathers retanned with resins, the new method gives a formaldehyde content stable over time by avoiding resin hydrolysis. 

### 2.5. Another Alternative to the Standard: Analysis by FIA with On-Line Acetylacetone Derivatization 

Another solution to avoid the delay time between derivatization and analysis (and the subsequent hydrolysis of resins co-extracted), could be an online derivatization. An online derivatization should be almost immediate, what is the case for the DNPH derivatization in harsh acidic conditions. However, such harsh acidic conditions are hardly compatible, in terms of chemical resistance of the pre-column reactor and tubing. Derivatization with acetylacetone (used in Part 2 of the standard) should be more adapted since the derivatization is implemented in mild conditions (at pH around 6) [19]. Furthermore, the absorption wavelengths of acetylacetone (412 nm) differs from the one of its derivatives with formaldehyde (295 nm). Thus, no chromatographic separation is more required provided that no dyes trigger interferences [12]. An on-line flow injection analysis set-up with on-line derivatization was home-designed. The flow injection analysis (FIA) with on-line derivatization was implemented with a LC-2040C Shimadzu system (without column). The FIA solvent (water/acetonitrile, 15/85 *v*/*v*) flow rate was set at 0.25 mL/min; the sample was injected (50 µL) via the autosampler and then on-line derivatized with an acetylacetone solution pumped at 0.25 mL/min. After the mixing in a zero-dead volume mixing tee, the reaction occurred in a 0.5 mL reactor (PTFE tube 0.25 mm i.d., 10 m in length), heated in an oven (column oven CTO-6A Shimadzu) at 90 °C. It corresponds to a derivatization duration of 1 min (the relative flow rates and the temperature were optimized to maximize the signal to noise ratio). Finally, the UV detection was achieved in the UV detector (cell volume 6 µL) at 412 nm. 

Because there is no delay between the end of the derivatization and the analysis, the resin hydrolysis should not occur during the derivatization. The three leathers studied did not have dyes that could generate interferences with acetylacetone derivatization. They were extracted according to the same protocol. 

**Figure 7:**
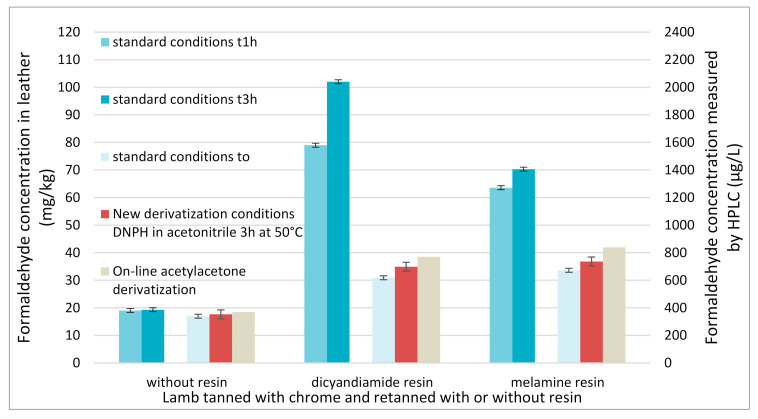
Comparison of three derivatization methods to determine formaldehyde in leathers: EN ISO 17226-1 standard conditions directly after the beginning of the derivatization reaction (in light blue), derivatization with DNPH in acetonitrile 3 h in a water bath at 50 °C (red) and acetylacetone derivatization with an online montage (in beige). For the standard conditions, the formaldehyde concentration analyzed during the time laps of the standard: in blue for 1 h and in dark blue for 3 h after the beginning of the derivatization reaction. Uncertainties given as two standard deviations (calculated with three measurements: three independent derivatizations).

Figure 7 shows that for leather retanned without any resin, all the derivatization methods (the standard one, the method with modified derivatization conditions or modified time frame and the FIA method) lead to comparable results (about 20 mg of free formaldehyde per kg of leather) and can be indifferently used. It also demonstrates that the FIA method with on line derivatization is a viable alternative for the automated determination of the formaldehyde content of leathers free of dyes. The total analysis time of the FIA method, from the automated injection by the autosampler of the chromatographic system to the UV detection, is less than 2 min, thus allowing the analysis of 30 samples per hour.

All these methods were then applied to quantify formaldehyde in leathers retanned with resins (dicyandiamide and melamine ones). The same extract of each leather was used for all the methods using DNPH derivatization whereas another extract of the same leathers was used for the FIA method with acetylacetone. The obtained results clearly show that the FIA method, the standard conditions with an immediate analysis and the new derivatization conditions lead to comparable results. This different extract could explain the little deviation obtained with the acetylacetone derivatization. In order for the reaction to be quick enough, a high temperature in the reactor is needed, and this high temperature can hydrolyse resin according to the literature [12].

## 3. Experimental Procedures

### 3.1. Leather Samples and Resin Samples

Leather samples were prepared in the tannery of the French Technical Center for Leather, footwear and leather goods (CTC, Centre Technique Cuir, Chaussure, Maroquinerie, Ganterie). Three lamb leathers were tanned with chromium. Then, the first leather (A) was retanned without resin, the second one (B) was retanned with 6% dicyandiamide resin and the third one (C) with 6% melamine resin (6 kg of resin for 100 kg of squeeze skin leather). For confidential reasons, resin suppliers are not mentioned.

### 3.2. Chemicals

Water was produced by a Milli-Q Ultra-pure water purification system (Merck KGaA, Darmstadt, Germany). For extraction: dodecane-1-sulfonic-acid sodium salt was obtained from Supelco (Merck KGaA, Darmstadt, Germany). For DNPH derivatization: 2,4-dinitrophenylhydrazine (DNPH) was bought from Supelco (Merck KGaA, Darmstadt, Germany), orthophosphoric acid 85+% and hydrochloric acid 37% were purchased from Fisher Chemical (Thermo Fisher Scientific, Illkirch-Graffenstaden, France), glacial acetic acid and sodium hydroxide were obtained from VWR (Fontenay-sous-Bois, France) and citric acid and sodium acetate were bought from Merck KGaA, Darmstadt, Germany.

For acetylacetone derivatization: acetylacetone was purchased from Fluka (Thermo Fisher Scientific, Illkirch-Graffenstaden, France), glacial acetic acid, and ammonium acetate were obtained from VWR (Fontenay-sous-Bois, France).

For chromatography: Acetonitrile (HiperSolv gradient grade) was purchased from VWR (Fontenay-sous-Bois, France), quality controls and calibration are made with a solution of formaldehyde 100 µg/mL in water purchased from AccuStandard (Techlab, Metz, France) and with a formaldehyde-2,4-DNPH solution 100 μg/mL in acetonitrile purchased from Supelco (Merck).

Buffers solutions, were prepared at 2 mol L^−1^. Orthophosphoric acid 85% and citric acid were respectively used for the preparation of phosphate solution (pKa = 2.12) and citrate solution (pKa = 3.13). For each buffer solution a suitable amount of sodium hydroxide was added to reach pKa. For the acetate buffer solution (pKa = 4.75) glacial acetic acid and sodium acetate were mixed to reach the desired pH. 

### 3.3. Sample Preparation and Extraction (Leather Extracts or Resin Extracts)

Leather samples were manually cut into small pieces (from 3 mm to 5 mm side length) in accordance with ISO 4044. Then, 2 g of leather pieces were weighed. For resin samples, 0.04 g of resin powder were weighed. The same extraction procedure was used for all the samples. This extraction is based on EN ISO 17226-1 standard. 50 mL of 0.1% dodecane-1-sulfonic-acid aqueous solution were added and samples were linear stirred in a water bath at 40 ± 1 °C for 60 ± 2 min. Then, leather extracts (or resin extracts) were immediately filtered on a glass fibre filter, cooled down to room temperature and derivatized.

### 3.4. DNPH Derivatization and Chromatographic Separation of DNPH Derivatives

Derivatization: The derivatization with DNPH was realized off-line, before the chromatographic separation. The DNPH solution was prepared by dissolving 0.06 g of DNPH either in 20 mL of orthophosphoric acid (for standard conditions) or in acetonitrile for buffered conditions.

A 10 mL derivatization flask was filled with 5 mL of leather extract, 4 mL of acetonitrile, 500 µL of DNPH solution and filled up to the mark with water. For buffered conditions, 500 µL of buffer solution were also added to reach a final buffer concentration of 0.1 mol L^−1^ in the flask. For each derivatization condition, a derivatization blank was run alongside the sample. For derivatization blank preparation, the leather extract was replaced by 5 mL of the extraction medium (0.1% dodecane-1-sulfonic-acid aqueous solution). Whatever the derivatization conditions, no artefact peaks were detected in the time range of formaldehyde. For quality controls, the leather extract was replaced by 5 mL of a formaldehyde standard solution prepared in the extraction medium (0.1% dodecane-1-sulfonic-acid aqueous solution). For experiments with resins, 5 mL of resin extract were used. The derivatization flasks were briefly shaken by hand. They were analysed after a given period of time as specified in the text. 

Chromatographic analysis: The separation and quantification of DNPH derivatives were implemented in reversed phase chromatography. Analyses were realized with an Agilent Series 1290 Infinity UPLC and a 1290 Infinity Diode Array Detector (DAD). The software was OpenLab CDS ChemSation Rev C.01.03. 

Analyses were realized with a ZORBAX Eclipse Plus C18 (Agilent) 3 × 100 mm (d_p_ = 1.8 μm) column, at 35 °C, with an acetonitrile/water mobile phase (from 35/65 *v/v* to 66/34 *v/v* in 3 min, and to 90/10 *v*/*v* in 0.5 min), at a flow rate of 1 mL min^−1^ and with an injection volume of 10 µL. The formaldehyde derivative was detected by UV at 350 nm with a reference wavelength of 580 nm. The calibration solutions (from 50 µg/L to 4000 µg/L) were prepared by diluting the commercial formaldehyde-2,4-DNPH solution (100 μg/mL) with a mixture of 50/50 acetonitrile/water. In the chromatograms obtained the first peak (at 1.2 min) is the residual derivatization reagent (DNPH). Formaldehyde-2,4-DNPH standard gave a reference retention time (2.1 min) and allowed to register the formaldehyde derivative spectra at 350 nm in the library. Formaldehyde derivative in samples were identified by comparison of retention time and UV spectra with formaldehyde derivative reference retention time and UV spectra in the library. Limit of detection (LOD) and limit of quantification (LOQ) (respectively 17 µg L^−1^ and 50 µg L^−1^) were determined according to NF T90-210 standard. Given the sample preparation conditions, these two values correspond to a LOD of 0.9 mg kg^−1^ and to a LOQ of 2.5 mg kg^−1^. The repeatability of the new derivatization method has been evaluated with 3 replicates (3 independent derivatizations). The relative standard deviation is less than 4%.

### 3.5. Flow Injection Analysis (FIA) with On-Line Derivatization with Acetylacetone

The flow injection analysis (FIA) with on-line derivatization was implemented with a LC-2040C Shimadzu system (without column). The FIA solvent was a water/acetonitrile mixture (15/85, *v*/*v*). The flow rate was set at 0.25 mL/min; the sample was injected (50 µL) via the autosampler and then on-line derivatized with an acetylacetone solution (0.01 M acetylacetone, 0.025 M glacial acetic acid and 1 M ammonium acetate) pumped at 0.25 mL/min. The mixing of the eluent and derivatization solution was implemented in a zero-dead volume mixing tee and then the reaction occurred in a 0.5 mL reactor (PTFE tube 0.25 mm i.d., 10 m in length), heated in an oven (column oven CTO-6A Shimadzu) at 90 °C. It corresponds to a derivatization duration of 1 min. Finally, the UV detection was achieved in the UV detector (cell volume 6 µL) at 412 nm (in LC-2040C Shimadzu). Calibration solutions were prepared from 50 µg/L to 4000 µg/L by diluting the commercial formaldehyde solution at 100 µg/mL with detergent solution (0.1% dodecane-1-sulfonic-acid sodium salt).

## 4. Conclusions

First, we have demonstrated that the EN ISO 17226-1 standard is not adapted to leather samples retanned with resins likely to release formaldehyde by hydrolysis. Indeed, we have shown that the formaldehyde content of leathers retanned with resins is largely overestimated according to this standard. This overestimation is due the concomitant hydrolysis of resins (in harsh acidic conditions) that releases formaldehyde during the derivatization step and during the waiting time on the autosampler before chromatographic analysis (the higher the waiting time, the higher the formaldehyde content measured).

A thorough study of the derivatization step has shown that replacing orthophosphoric acid with less acidic buffer solutions is not enough to avoid hydrolysis. Alternative derivatization conditions (3 h at 50 °C in a water bath) with DNPH solubilized in acetonitrile (instead of orthophosphoric acid) would allow a complete derivatization of formaldehyde while avoiding the hydrolysis of co-extracted dicyandiamide and melamine resins. Moreover, the as-obtained leather derivatized extracts are stable over time and their analysis can be delayed in time without any restriction. An alternative method is also proposed (for leathers containing no dyes) with a home-designed FIA method with on-line derivatization with acetylacetone and UV detection. Formaldehyde contents determined with this FIA method agree with the formaldehyde content measured immediately at the end of derivatization reaction in standard conditions or with the formaldehyde content measured with the new derivatization conditions.

Given that the proposed modification of the derivatization step avoids the problem of the hydrolysis of resins in the HPLC determination of formaldehyde, this upgrade should be disseminated beyond the leather sector and support textile testing. This is more important given that the ECHA has recently modified the ANNEX XVII TO REACH to more severely restrict formaldehyde content in textile garments.

## Figures and Tables

**Table 1:** pH, concentration, DNPH derivatization reaction time and temperature depending on buffer solutions added.

Buffer Solution	Buffer Solution pH (pH = pKa)	Final Buffer Concentration (mol/L)	Time Necessary to Have a Complete Reaction	Temperature (°C)
Phosphate	2.12	0.1	1 h 30	Room temperature
Citrate	3.13	0.1	1 h	Room temperature
Acetate	4.75	0.1	1 h 30	Room temperature
Without buffer	-	-	3 days	Room temperature
Without buffer	-	-	3 h	50 °C
